# Testing of aerosolized ciprofloxacin nanocarriers on cystic fibrosis airway cells infected with *P. aeruginosa* biofilms

**DOI:** 10.1007/s13346-021-01002-8

**Published:** 2021-05-28

**Authors:** Jenny Juntke, Xabier Murgia, Nazende Günday Türeli, Akif Emre Türeli, Chelsea R. Thorn, Marc Schneider, Nicole Schneider-Daum, Cristiane de Souza Carvalho-Wodarz, Claus-Michael Lehr

**Affiliations:** 1 Helmholtz Institute for Pharmaceutical Research Saarland (HIPS), Helmholtz Center for Infection Research (HZI), Saarbrücken, Germany; 2 MyBiotech GmbH, Industriestr. 1B, Überherrn, Germany; 3 Department of Pharmacy, Saarland University, Saarbrücken, Germany; 4 University of South Australia, Clinical and Health Science, Adelaide 5000, Australia

**Keywords:** In vitro model, Pulmonary drug delivery, Nanoparticles, Cystic fibrosis, Aerosol

## Abstract

The major pathogen found in the lungs of adult cystic fibrosis (CF) patients is *Pseudomonas aeruginosa*, which builds antibiotic-resistant biofilms. Pulmonary delivery of antibiotics by inhalation has already been proved advantageous in the clinic, but the development of novel anti-infective aerosol medicines is complex and could benefit from adequate in vitro test systems. This work describes the first in vitro model of human bronchial epithelial cells cultivated at the air–liquid interface (ALI) and infected with *P. aeruginosa* biofilm and its application to demonstrate the safety and efficacy of aerosolized anti-infective nanocarriers. Such a model may facilitate the translation of novel therapeutic modalities into the clinic, reducing animal experiments and the associated problems of species differences. A preformed biofilm of *P. aeruginosa* PAO1 was transferred to filter-grown monolayers of the human CF cell line (CFBE41o-) at ALI and additionally supplemented with human tracheobronchial mucus. This experimental protocol provides an appropriate time window to deposit aerosolized ciprofloxacin-loaded nanocarriers at the ALI. When applied 1 h post-infection, the nanocarriers eradicated all planktonic bacteria and reduced the biofilm fraction of the pathogen by log 6, while CFBE41o- viability and barrier properties were maintained. The here described complex in vitro model approach may open new avenues for preclinical safety and efficacy testing of aerosol medicines against *P. aeruginosa* lung infection.

**Supplementary Information:**

The online version contains supplementary material available at 10.1007/s13346-021-01002-8.

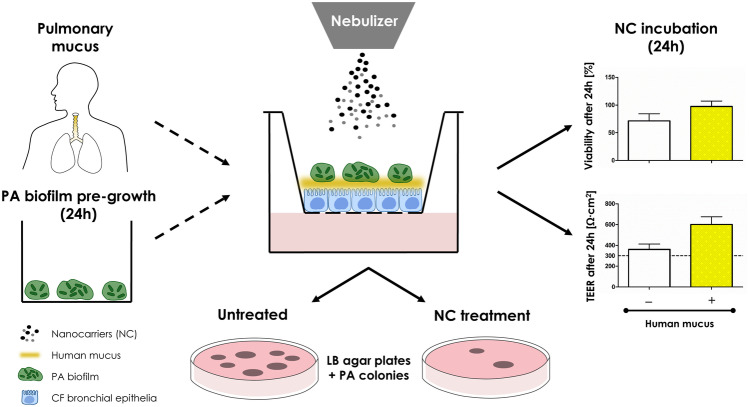

## Introduction

The genetic disease cystic fibrosis (CF) currently affects more than 70,000 people worldwide [1]. CF patients usually suffer from thick and sticky mucus accumulating in their lungs, favoring bacterial growth, and leading to persistent and recurrent lung infections [2]. These infections are the major cause of morbidity and mortality in patients [3]. Of adult CF patients, 80% are chronically infected with the pathogen *Pseudomonas aeruginosa* [4, 5]. Despite aggressive and intensive treatment, *P. aeruginosa* builds antibiotic-resistant biofilms, leading to treatment failure [6, 7]. Biofilms are bacterial communities where bacteria are aggregated or attached to a surface (e.g., mucosal epithelium) [8], enclosed by a self-produced matrix of exopolysaccharides, DNA, and proteins—the so-called extracellular polymeric substances. Bacteria living in such a biofilm are less susceptible to anti-infectives than those growing in planktonic form. Antibiotics usually target aerobically growing pathogens or actively dividing bacteria, like aminoglycosides or ß-lactam antibiotics, respectively. In biofilms, especially in the center, *P. aeruginosa* has an anaerobic growth and slow cell division rates, resulting in limited antibiotic efficiency [9, 10]. Another major problem in CF treatment is the biological barrier formed by mucus and biofilm polymers, leading to poor antibiotic penetration, low diffusion rates of the antibiotic into the biofilm [11–15], and inactivation of the drug by biofilm components [16].

To minimize systemic exposure and maximize local drug concentration in the lungs, antibiotic therapy may be best administered as inhalable aerosols [17, 18]. While pulmonary delivery of antibiotic drugs per see is well established, their efficacy can be further improved in combination with nanotechnology [19]. In contrast to the “naked” molecule, nanocarriers hold the promise to get entrapped by bacterial biofilms and thus provide a platform for the controlled release of anti-infective agents immediately at the site of action. Sophisticated particle surface engineering may further improve biofilm penetration and interaction with its components [16, 20, 21].

Existing in vivo models of CF-associated lung infections typically use mice [22, 23] or other rodents [24, 25] for testing the safety and efficacy of specific treatments. Unfortunately, these models poorly reflect the human situation, leading to failures in clinical trials. Also, for ethical reasons, alternative testing methods to replace, reduce, and refine such animal experiments according to the 3R principle of Russell and Burch [26] are needed. Current in vitro models for testing antibiotics usually rely on biofilms grown on non-living surfaces [27, 28]; however, such biofilms differ significantly from those found in CF patients [29].

Moreover, such models do not allow for monitoring (patho)physiological changes of the epithelium (e.g., epithelial barrier function, cellular viability) under the influence of the infection and some anti-infective therapy. Co-culture models of *P. aeruginosa* biofilms cultivated on living human airway cells have already been described. However, due to rapid bacterial overgrowth, these cell cultures were not viable for more than 8 h [30, 31]. Even though the epithelial cells from these studies were cultivated at the air–liquid interface (ALI) initially, cells were later maintained under submerged conditions. Therefore, these bacterial-epithelial co-cultures are not suitable for testing aerosolized medicines, which also requires ALI conditions.

Furthermore, the lack of mucus in these models was also not addressed. However, as we have already shown, the susceptibility of *P. aeruginosa* to antibiotics is reduced when they form a biofilm in the presence of mucus [32]. Thus, a human-based in vitro system with the potential to predict and thus further improve antibiotic delivery to bacteria residing at the lung’s air-blood barrier is still in need.

This study aimed to address the above shortcomings of existing in vitro models to improve the preclinical tools for testing aerosolized anti-infective nanocarriers. We here describe a complex in vitro model consisting of filter-grown monolayers of human bronchial epithelial cells, supplemented with human trachea-bronchial mucus and infected with preformed *P. aeruginosa* biofilms under ALI conditions. This model was found to be suitable for testing the safety and efficacy of aerosolized antibiotic nanocarriers. Apart from ethical advantages by complying with the abovementioned 3R principle, replacing the routinely used but poorly predictive animal models with complex in vitro models (CIVM’s), mainly when based on human cells, also holds the critical perspective to enable the more successful and faster translation of novel anti-infective therapies into the clinic.

## Material

CFBE41o- cells were received as a kind gift from Dr. Dieter C. Gruenert (University of California, San Francisco, CA, USA). Calu-3 cells (clone HTB-55) and the fluorescent-labeled PAO1 strain (ATCC® 10145GFP™) were obtained from American Type Culture Collection (ATCC; Rockville, MD, USA).

Minimal essential medium (MEM), non-essential amino acids (NEAA), sodium pyruvate, and Hank’s balanced salt solution (HBSS) were purchased from Gibco™ (Thermo Fisher Scientific Inc., Waltham, MA, USA). Ampicillin was acquired from Carl Roth (Karlsruhe, Germany) and fetal calf serum (FCS) from Lonza (Basel, Switzerland). The 12-well plate Transwell® inserts, 0.4 µm pore size, were procured from Corning (Cat. No. 3460, Corning™ Costar™, Lowell, USA). Ciprofloxacin base, dimethyl sulfoxide (DMSO), the non-ionic surfactant, a Poloxamer 188 (Pluronic F-68), sodium dodecyl sulfate (SDS), phosphate-buffered saline (PBS), glucose, LB broth, arginine, MTT (3-(4,5-dimethylthiazol-2-yl)-2,5-diphenyltetrazolium bromide), and Triton X-100 were purchased from Sigma-Aldrich (Munich, Germany). PLGA (poly(lactic-co-glycolic) acid was bought from PCAS (Longjumeau Cedex, France); Pantotile® CIPRO eardrops were provided from Pierre Fabre Pharma GmbH (Freiburg, Germany). All solvents used for nanocarrier preparation were of analytical grade and were obtained from VWR (Darmstadt, Germany).

## Methods

### Nanocarriers: preparation and nebulization

PLGA (poly(lactic-co-glycolic) acid) particles were prepared with the MicroJet Reactor (MJR), a technology of impinging jets in a micron-sized chamber [33], which produces nanocarriers under controlled conditions. Through controlling process parameters, e.g., flow rate, temperature, and gas pressure in the chamber, the nanocarrier size can be optimized. An SDS-ciprofloxacin complex was formed to overcome the low solubility of ciprofloxacin [34]. This complex, dissolved in a DMSO solution containing PLGA (solvent system) and Pluronic F-68 solution as the non-solvent system, was loaded into the MJR (Synthesechemie, Heusweiler, Germany).

The nanocarriers’ size was determined by dynamic light scattering using a ZetaSizer ZS90 (Malvern, UK) after 1:100 dilution in distilled water. After the precipitation process, the particles’ purification was achieved by dialysis with D-Tube™ with an MWCO for 6–8 kDa (Merck Millipore, Darmstadt, Germany).

Drug loading was calculated based on the drug amount determined by HPLC (Waters Symmetry C18 column at 30 °C with isocratic elution mode using 25 mM phosphoric acid pH = 3 adjusted with triethylamine/acetonitrile (83:13 *v*/*v*) at a flow rate of 1.5 ml/min. The drug was detected at λ = 278 nm [34]) from freeze-dried particles dissolved in DMSO and was reported as the amount of ciprofloxacin in percent relative to the weighed amount of formulation. Post-preparation nanocarriers were further lyophilized by freeze-drying using an Alpha 2–4 LSCplus freeze dryer (Martin Christ Gerfriertrocknungsanlagen GmbH, Osterode am Harz, Germany) [33].

### Analysis of drug-loaded nanoparticles upon nebulization

Nanocarriers were suspended in Krebs ringer buffer (KRB, NaCl 142.03 mM, KCl 2.95 mM, K_2_HPO_4_·3H_2_O 1.49 mM, HEPES 10.07 mM, D-glucose 4.00 mM, MgCl_2_·6H_2_O 1.18 mM, CaCl_2_·2H_2_O 4.22 mM; pH 7.4) supplemented with 10% FCS to a final concentration of 350 µM (ciprofloxacin equivalent), a concentration previously determined to be non-toxic to the bronchial cell lines [35].

Nanocarriers were then nebulized with an Aerogen Solo chamber attached to the Aeroneb® lab micropump control unit (INSPIRATION Medical GmbH, Bochum, Germany) [36]. To assure the nanosuspension’s quality, the particle size of the resuspended lyoproduct was measured before and after nebulization by dynamic light scattering (Zetasizer Nano ZS, Malvern Instruments, Malvern, UK). In preliminary experiments, 200 µl of antibiotic-loaded nanocarrier suspension with a concentration of 350 µM was nebulized into empty wells of a 12-well plate. The aerosol was recovered by pipetting the contents of each well into Eppendorf® tubes. Samples were diluted 1:100, and the drug content was measured with a UHPLC method (Dionex Ultimate® 3000 UHPLC) with an Accucore column (RP 18, 150 mm × 2.1 mm, 2.6 µm, Thermo Fisher Scientific Co., Waltham, MA, USA).

### Cell culture of bronchial epithelial cells

#### CFBE41o-

The CFBE41o- cells are derived from a cystic fibrosis patient and homozygous for ΔF508 mutation [37, 38]. For standard cultivation, the cells were maintained in MEM supplemented with 10% FCS, 1% NEAA, and 600 mg/l glucose at 5% CO_2_ atmosphere and 37 °C, respectively. Cells were fed every 2–3 days and passaged weekly.

For the infection experiments, the CFBE41o- cells were seeded with a density of 50,000 cells/well in 12-well Transwell® plates with a pore size of 0.4 µm.

#### Calu-3

The human bronchial epithelial cell line Calu-3 HTB-55 (ATCC, passage 31–51) was also used as a model cell line for the in vitro model. The cells were maintained in MEM with 10% FCS, 1% NEAA, and 1% sodium pyruvate at 5% CO_2_ atmosphere and 37 °C. Cells were fed every other day and passaged weekly. For infection experiments, cells were seeded with a density of 100,000 cells/well in 12-well Transwell® plates with a pore size of 0.4 µm.

CFBE41o- or Calu-3 cells were lifted to ALI 2–3 days after seeding on Transwell® inserts and fed every other day until forming a confluent monolayer, and barrier properties exceeded 300 Ω*cm^2^ (i.e., 7 days for CFBE41o- or 10–12 days for Calu-3 cells). Both cells were used for up to 20 passages and checked for mycoplasma contamination every 2 months, with the MycoAlert™ mycoplasma detection kit (LONZA), following the manufacturer’s instructions.

### Mucus preparation and application

Since CFBE41o- cells do not produce mucus [39, 40]; freeze-dried human tracheobronchial mucus was applied to cells 5 days after seeding. Human mucus collected from endotracheal tubes (in compliance with a protocol approved by the Ethics Commission of The Chamber of Medicine Doctors of the Saarland, file number 19/15) from patients undergoing surgery not related to pulmonary conditions were pooled and freeze-dried to disks on suitable templates. Details of this protocol are described elsewhere [32, 41]. Mucus disks were carefully removed from the template, placed on top of CFBE41o- monolayers, and rehydrated with 50 µl of cell culture medium. Transwell® plates with CFBE41o- cells and mucus were incubated at 37 °C, at 150 rpm for 24 h to allow an even distribution of the mucus.

### Measurement of transepithelial electrical resistance

To determine the barrier properties, cell monolayers were incubated with 500 µl apical and 1500 µl basolateral medium for 30 min at 37 °C, and TEER was measured with an epithelial voltohmmeter (EVOM, World Precision Instruments, Sarasota, FL, USA) equipped with STX2 “chopstick” electrodes. Values were corrected for background value (cell-free Transwell® filter with the medium). After the measurement, ALI conditions were restored by removing adequate amounts of medium from both compartments.

### Bacterial strains and culture conditions

*P. aeruginosa* strain PAO1-WT (wild type) [42] and the fluorescent-labeled strain ATCC® 10145GFP™ were grown in LB broth, which was supplemented with 300 µg/ml ampicillin for the GFP (green fluorescent protein) strain. The GFP-labeled strain was used for CLSM images. For all other readouts (i.e., TEER, MTT, CFU measurement, scanning electron microscopy), the WT strain was used.

Overnight cultures were grown by inoculating 10 ml of LB broth with a single colony of bacteria and incubating the broth for 16–18 h at 37 °C and 180 rpm.

For biofilm growth, an overnight culture of PAO1 in the exponential growth phase was washed and diluted to an OD_600_ 0.1 (colony-forming unit (CFU) ~ 5 × 10^7^/ml). Then, 15 µl of this dilution was inoculated into the minimal medium M63 (M63, supplemented with 0.2% glycerol, 0.4% arginine, and 1 M MgSO_4_) to a final volume of 200 µl in 24-well plates. Cultures were then incubated under static conditions at 37 °C for 24 h to allow biofilm formation. Biofilm formation was assessed with crystal violet staining according to protocols described elsewhere [43, 44], with slight adaptation as described below. The GFP and WT strains showed similar biofilm growth under the conditions described above (data not shown).

### Co-culture of bronchial cells and *P. aeruginosa* PAO1

CFBE41o- or Calu-3 cells were used for the infection experiment, respectively, after 7 or 12 days of seeding. The cultures at ALI condition were infected with a preformed biofilm of PAO1 (CFU/ml of biofilm ~ 4.6 × 10^8^). The biofilms (pre-grown for 24 h in a 24-well plate) were gently scratched off and transferred with a pipette to the apical side of the epithelial cells. Biofilms were allowed to attach to cell monolayers for 1 h. After that time, unattached bacteria and excess medium were removed from the apical side, and the basolateral medium changed to MEM, which was supplemented with 0.4% arginine. TEER measurement was assessed as described before. Biofilm formation, epithelial cell viability, and bacteria survival were further determined after 4 and 24 h infection, as described below. During the experiment, the epithelial cell monolayers’ integrity was checked with a light microscope (ZEISS, Vert.A1).

### Evaluation of biofilm with crystal violet

To assess biofilm biomass before and after the biofilm transference, crystal violet staining was performed. Briefly, the biofilm was heat fixed in the well plate at 60 °C for 1 h and subsequently stained with 0.1% (*w*/*v*) crystal violet for 15 min. Thereafter, the excess dye was removed with deionized water, leaving behind the stained fixed biofilm. Solubilization with 30% (*w*/*v*) acetic acid enabled quantification of total biofilm biomass by reading the absorbance of crystal violet at 595 nm (Inspire Multimode Plate reader, Perkin Elmer, Waltham, MA).

### Efficacy testing of antibiotic-loaded nanocarriers against planktonic and biofilm *P. aeruginosa*

Two treatment time points were chosen for this study. The suspended nanocarriers were nebulized on the co-culture 1 h or 4 h after the infection. As a negative control, infected cells were treated only with KRB at the 1 h time point. The positive control was uninfected cells treated with the nanosuspension at the 1 h time point. Panotile® CIPRO eardrops were chosen as a reference product to compare the antibiotic-loaded nanocarriers to a commercially available ciprofloxacin product; 1 ml of the aqueous Panotile solution contains 2.32 mg ciprofloxacin hydrochloride equal to 2 mg/ml ciprofloxacin. Additional ingredients are glycerol, polysorbate 20, sodium acetate, acetic acid 99%, methylcellulose, hydrochloric acid, sodium hydroxide, and water for injection. The Panotile® CIPRO eardrops were diluted with KRB (with 10% FCS) to the appropriate concentration (350 µM ciprofloxacin) and nebulized onto the infected epithelial cells at one of these time points in the same manner as the nanocarriers. The efficacy of either treatment was determined by measuring the cell viability (MTT assay) and barrier properties (TEER). Furthermore, the CFU of planktonic and biofilm-bound bacteria was assessed in separate experiments after these different treatment options.

### Measurement of barrier properties (TEER) and cellular viability (MTT)

The basolateral medium was removed as well, and both compartments were replenished with HBSS (500/1500 µl) for 30 min to obtain the TEER values. ALI conditions were restored, and cell viability was determined via MTT assay. The MTT assay was performed by following the manufacturer’s instructions. Briefly, the cells were washed and incubated with 300 µl MTT solution (500 µg/ml) apical and 500 µl HBSS basolateral for 4 h at 37 °C and 5% CO_2_ atmosphere, protected from the light. Then, 250 µl of the apical solution was discarded, and 200 µl DMSO was added in each well to solubilize the resulting formazan crystals. The negative control for cell viability was obtained by incubating uninfected cells with 1% Triton X-100 in HBSS for 30 min. After 30-min incubation with MTT reagent, the samples’ supernatants were transferred to a 96-well plate and measured with a plate reader (TECAN, infinite M200 pro, Männedorf, Switzerland) at the absorption of 550 nm.

### Colony-forming unit assay

After 24-h infection, supernatants (resulting mainly from nebulized nanocarriers and medium transported from the basolateral to the apical side) were removed, serially diluted in PBS, and 0.1 ml of suitable dilution plated on agar plates. CFU counts resulting from cell supernatants are related to the planktonic bacteria, while the CFU counts resulting from bacteria adherent to epithelial cells are considered as biofilms. To lyse the epithelial cells, 500 µl of PBS with 0.1% Triton X-100 was added apically. The co-culture lysates were scraped from wells, vortexed, serially diluted, and plated on agar plates, as done for the supernatant. The plates were incubated overnight at 37 °C, and the CFU was determined the next day.

### Scanning electron microscopy 

The preparation of the samples was performed 24 h after infection and treatment. The cells were washed once with PBS at 37 °C, followed by fixation with glutaraldehyde 3% for 2 h at room temperature. Samples were then washed with PBS and dehydrated with a graded ethanol series (30–100%). Filters were cut from inserts and mounted on aluminum stubs equipped with a carbon disk and sputtered with gold (Quorum Q150R ES, Quorum Technologies Ltd, Laughton, UK). Images were taken with an EVO HD 15 microscope (acceleration voltage 5 kV; Software SmartSEM, Zeiss, Jena, Germany) under high vacuum conditions (3.8 × 10^6^ mbar in the sample chamber).

### Confocal laser scanning microscopy

For confocal images, the GFP-labeled strain *P. aeruginosa* PAO1-GFP was used to assess biofilm formation before and after transferring between 24-well plate and onto CFBE41o- monolayer and following treatments. Before cells were fixed with 3% paraformaldehyde solution and stained with DAPI (200 ng/ml, Life Technologies, Darmstadt, Germany), an additional propidium iodide (PI) staining (40 µg/ml PI in PBS) was conducted. Since PI can only enter cells and intercalate with nucleic DNA when their membrane is damaged, this staining allows distinguishing between dead cells (red nuclei, PI) and living cells (blue nuclei, DAPI). After staining, filters were cut from Transwell® inserts, mounted on glass slides, and covered with a coverslip. They were then examined with a Leica TCS SP8 with an AOBS beam splitter and a Leica HyD detector (Mannheim, Germany). Using the 25 × water immersion objective (Fluotar VISIR 25 × /0.95 water) or 63 × oil immersion objective, images were taken with a 1024 × 1024 resolution and processed with LAS X software (Leica).

### Statistical analysis

Data are presented as mean ± standard error of the mean (SEM) from at least 3 independent experiments. Statistical analysis was performed with the GraphPad Prism 5 software.

The change in TEER values before and after mucus addition and changes in crystal violet staining biofilm biomass before and after transferring were compared with a *t* test. TEER and CFU values after different treatment time points were compared with the one-way ANOVA and Bonferroni post-test. *P* values < 0.05 were considered significant as described in the respective figures legends.

## Results

### Nanoparticle characteristics

Nanocarriers with sizes of 236.7 ± 22.6 nm and a drug loading of 14% were found (Table 1, supplementary material). Suspending the particles in KRB supplemented with FCS led to an increase in particle size, to 374 ± 41 nm. Due to proteins in FCS, the formation of a corona could be observed. The nebulization process had no significant effect on the particle size (Table 1, supplementary material). After nebulization, the recovered suspension had a concentration of 234 ± 30 µM, which corresponds to a recovery rate of 67% (~ 140 µl), indicating an acceptable deposition efficacy (Table 2, supplementary material).

### Mucus enhances barrier properties of CFBE41o- cells and protects them during bacterial infection 

**Fig. 1:**
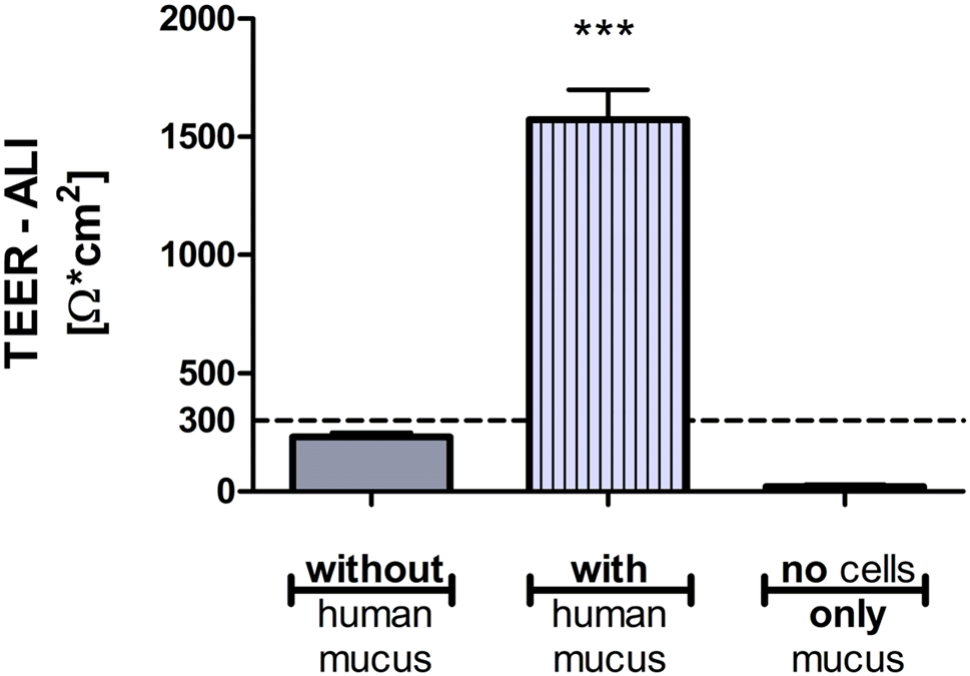
TEER values of CFBE41o- cells without or with additional human mucus. Without the addition of mucus, TEER did not reach 300 Ω*cm^2^ (solid gray bar). When human mucus was applied onto the cells, TEER values increased by sevenfold (striped bar). Mucus alone did not show barrier properties (right bar). The mean ± SEM from at least 3 independent experiments are shown with *n* = 18 wells (cells without mucus), *n* = 34 wells (cells with mucus), and *n* = 4 wells (only mucus). ***p < 0.0001

As mucus plays a significant role in CF chronic infections, a disk of freeze-dried human mucus was added to the apical compartment of the in vitro system. As we have previously shown, upon mucus hydration, both cell viability and mechanical mucus properties are preserved [41]. Measurement of TEER values 1 day after mucus application showed a sevenfold increase compared to control cells without mucus (1573 ± 126 Ω*cm^2^ vs. 230 ± 20 Ω*cm^2^; Fig. 1). TEER values of mucus alone (cell-free Transwell® with mucus only) reached 21 ± 8 Ω*cm^2^.

### Co-culture of CFBE41o- cells and PAO1 biofilm 

Direct infection of the epithelial cells with a multiplicity of infection (MOI) 1:20 of PAO1 did not lead to a stable model suitable for further testing. Even though earlier biofilms formed on the epithelial monolayer after 4 h with this approach, further cultivation for greater than 8 h without antibiotics led to the destruction of the cell monolayers and inevitable cell death (data not shown). Trials without the external human mucus layer led to an even faster death of the human cells. Thus, the approach to pre-grow the biofilm externally was taken, as described in the methods.

**Fig. 2:**
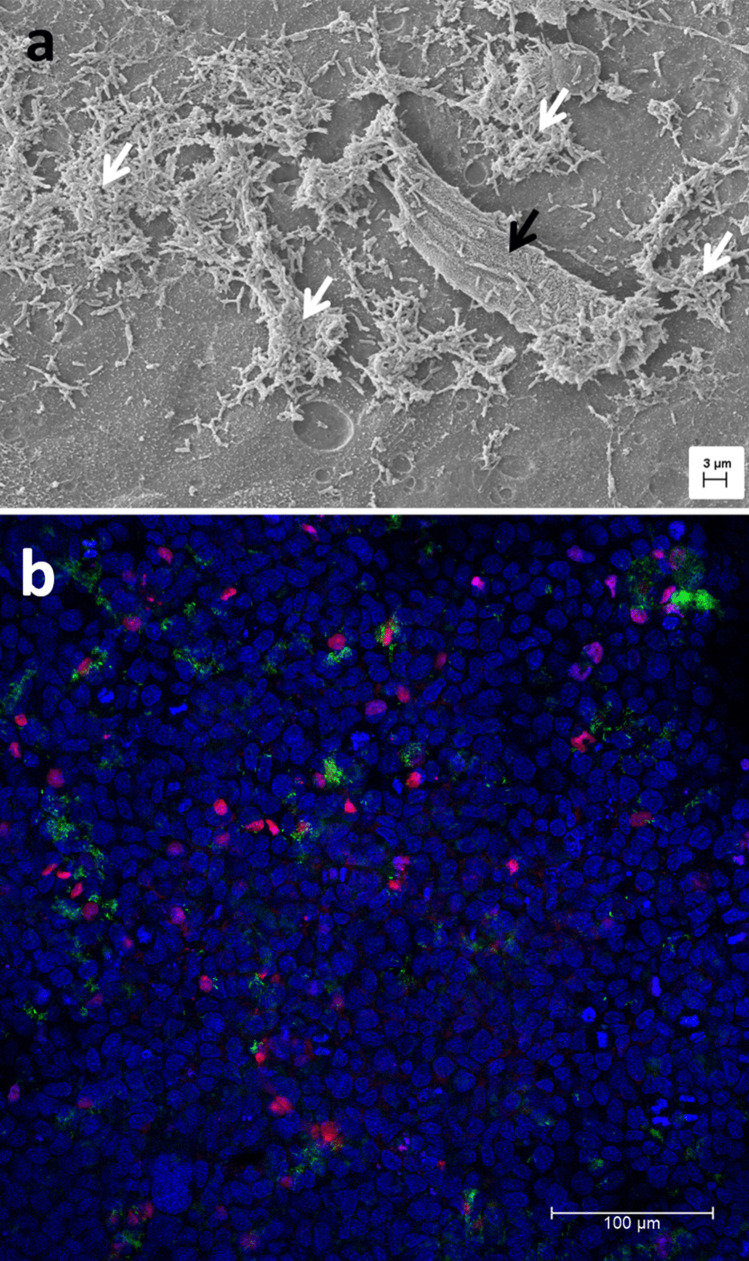
Images of the co-culture with fixation 1 h after infection**.** Scanning electron microscopy image (**a**) of intact CFBE41o- monolayer with mucus, planktonic bacteria, and biofilm on top. Black arrow indicates mucus; white arrows are biofilm fragments. The single bacteria can be considered planktonic. Confocal image (**b**) shows a confluent epithelial cell monolayer as indicated by a tight pattern of blue nuclei (DAPI). Dead cells are stained in red (propidium iodide). Green-labeled bacteria (GFP strain) form small clusters, which are only observed occasionally

**Fig. 3:**
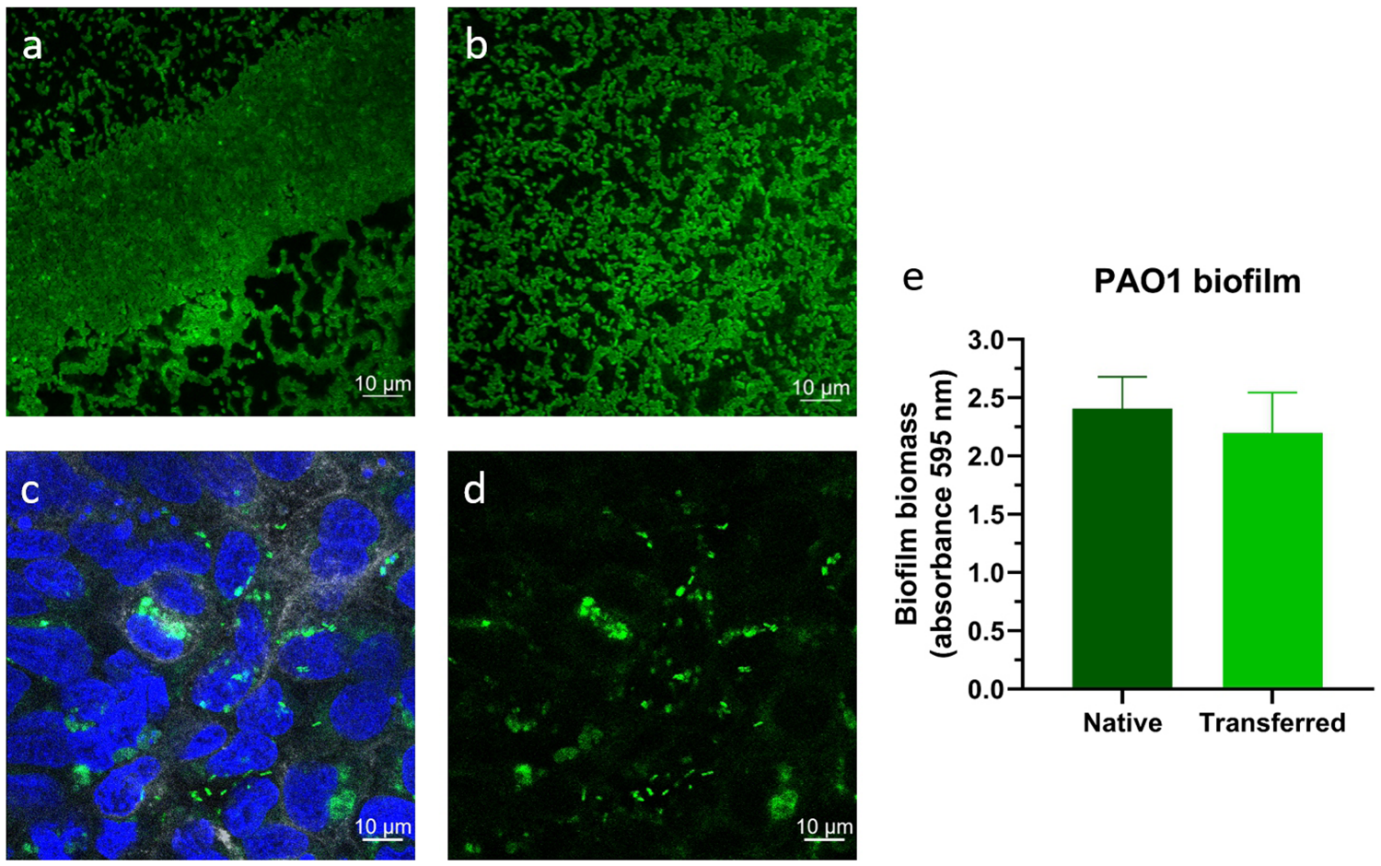
Confocal laser scanning micrographs of PAO1 GFP labeled (**a**) incubated in 24-well plate to form biofilm for 24 h, (**b**) scratched off and transferred into a new well after formation, (**c**) CFBE41o- cells stained with DAPI and incubated with PAO1 GFP-labeled biofilm after 4 h, and (**d**) is the GFP channel only of (**c**). (**e**) Crystal violet absorbance at 595 nm, indicating the biofilm biomass of the PAO1 biofilm grown in the 24-well plate for 24 h before (native) and after the transfer process (transferred), data represented as mean ± SEM from four independent experiments, *n* = 12. No statistical significance was assessed by an unpaired Student’s *t* test (*p* = 0.114)

Figure 2a shows a scanning electron microscopy micrograph of the CFBE41o- cells (with extra mucus layer) 1 h after infection with the preformed PAO1 biofilm. A tight monolayer of cells with microvilli-like structures and mucus on top (black arrow) is observed. The confocal micrograph in Fig. 2b also shows an intact monolayer of the CFBE41o- cells (tight pattern of blue nuclei) with few dead cells (red nuclei) 1 h after the infection and biofilm microcolonies; those are only visible in certain areas (white arrows in Fig. 2a, GFP bacteria in Fig. 2b) and do not cover the epithelial cells completely when transferred on top of the CFBE41o- cells and mucus. In Fig. 3, the native (preformed) 24 h PAO1 (labeled with GFP) biofilm (Fig. 3a) is compared to the transferred biofilm (Fig. 3b). While the transferred biofilm has smaller microcolonies than the native biofilm, quantification of the total biofilm biomass via crystal violet staining indicated no significant differences in the architectures before and after the biofilm is transferred into a new well (*P* = 0.114, Fig. 3e). The same applied to the PAO1 (wild type) biofilms, further suggesting that the biofilm’s integral structure does not change significantly. Moreover, upon being transferred onto the CFBE41o- cells alone, the biofilm retains microcolonies as observed in Fig. 3c, d. Also, single planktonic bacteria are present; yet, micro-colonies predominate 4 h post-infection (Fig. 3c, d).

Without antibiotic treatment, cells did not endure the preformed biofilm conditions for more than 4 h, leading to loss of barrier properties and subsequent cell death. However, transferring a preformed biofilm on top of the ALI-grown CFBE41o- cells allowed mimicking at least temporarily the situation of infected CF airways. Therefore, this approach was further pursued to evaluate aerosolized nanocarriers loaded with ciprofloxacin.

### Early treatment with antibiotic nanocarriers makes epithelial cells survive bacterial infection

Aerosolized nanocarriers or KRB buffer as negative control (no antibiotic treatment) were deposited onto the ALI co-culture at different time points. In a previous study, the chosen concentration of the nanocarriers (350 µM) was found to be non-toxic to the epithelial cells [35].

To evaluate the potential of such treatment to eradicate the PAO1 biofilm and preserve the epithelial cell monolayer’s integrity, viability assays, TEER measurement, scanning electron microscopy, and CLSM-imaging were used as read-outs.

**Fig. 4:**
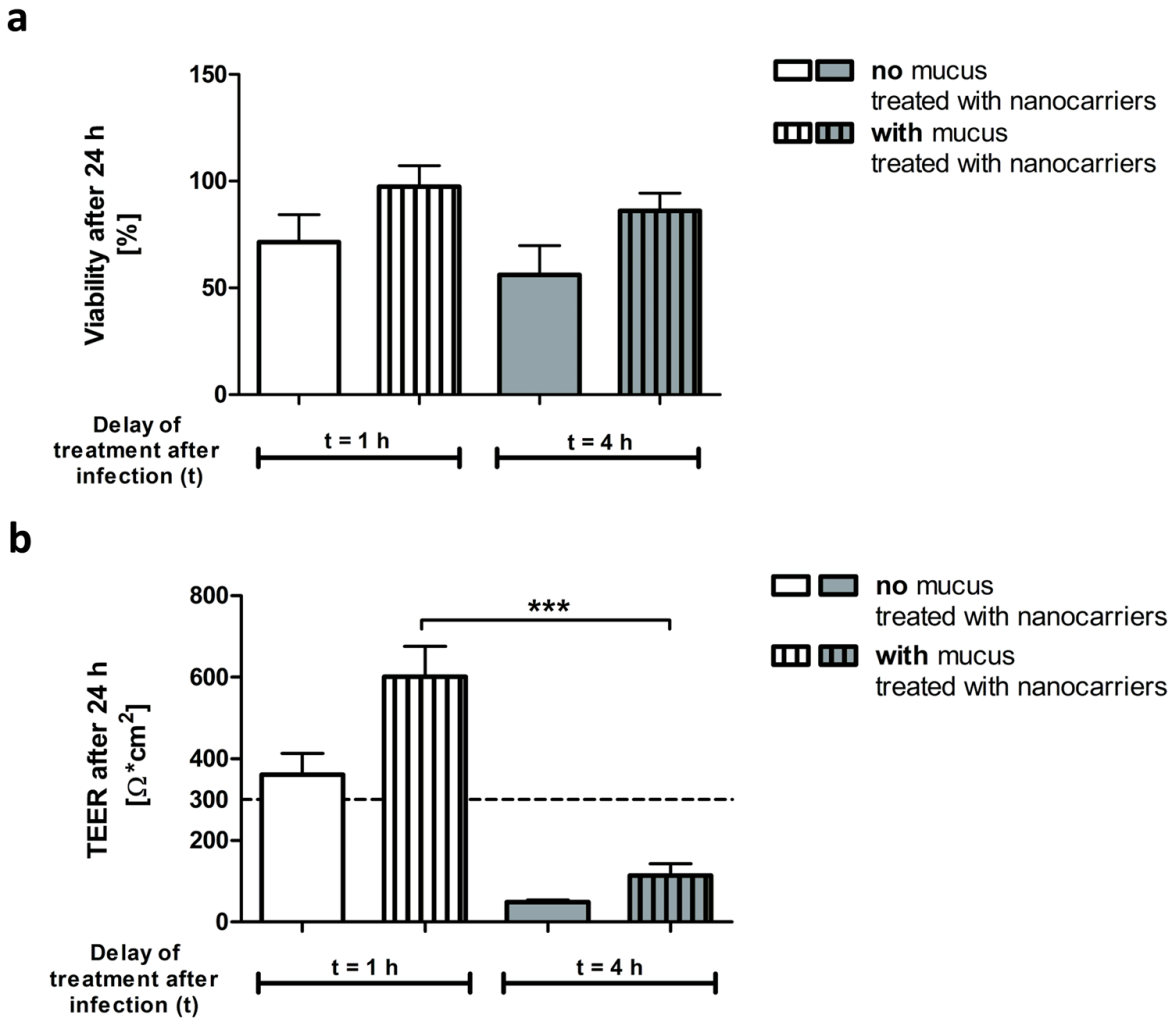
Viability and TEER of CFBE41o- cells after infection. Viability (**a**) of the CFBE41o cells (MTT assay). Early treatment (*t* = 1 h) with antibiotic-loaded nanocarriers helps the cells to survive the infection. Cells covered by mucus (striped bars) conserved their viability even when nanocarrier-treatment was performed at a later time point (*t* = 4 h). TEER (**b**) measurement after 24 h showed that with early treatment (t = 1 h), barrier properties of the CFBE41o- cells could be maintained. Treatment after 4 h resulted in a loss of barrier properties. Values are presented as mean ± SEM with *n* = 5 from 5 independent experiments (viability) and *n* ≥ 7 from ≥ 7 independent experiments (TEER). ***p < 0.0001

An MTT assay was performed 24 h after the infection. The viability of CFBE41o- cells infected with PAO1 biofilm for 1 h before treatment with the nanocarriers reduced to 65% (no mucus, Fig. 4a). When the antibiotic treatment was performed later (*t* = 4 h), the cell viability further decreased to 51%. However, in the presence of mucus, the cell viability after 1 h or 4 h infection improved to 93% and 80%, respectively, with the nanocarrier treatment.

TEER measurements demonstrated the protective role of human mucus in maintaining epithelial barrier function after infectious challenges (Fig. 4b). Cells that had received mucus before the infection showed almost twice the TEER values compared to the cells without human mucus (TEER 361 ± 52 Ω*cm^2^ versus 601 ± 75 Ω*cm^2^). This was at least the case when the antibiotic treatment occurred 1 h post-infection. When antibiotic-loaded nanocarriers were applied 4 h post-infection, the cells could not maintain their barrier properties even in the presence of mucus. However, TEER values of cells that had received mucus (TEER 115 ± 29 Ω*cm^2^) were still twice as high compared to cells without it (TEER 49 ± 5 Ω*cm^2^). Notably, the infected cell monolayers that had not received any antibiotic treatment (i.e., only blank KRB nebulized on the cells) hardly showed any remaining barrier function (TEER ~ 10 Ω*cm^2^, data not shown). In contrast, uninfected cells which received the nanocarriers showed high barrier function after 24 h (TEER ~ 1932 ± 167 Ω*cm^2^, data not shown).

**Fig. 5:**
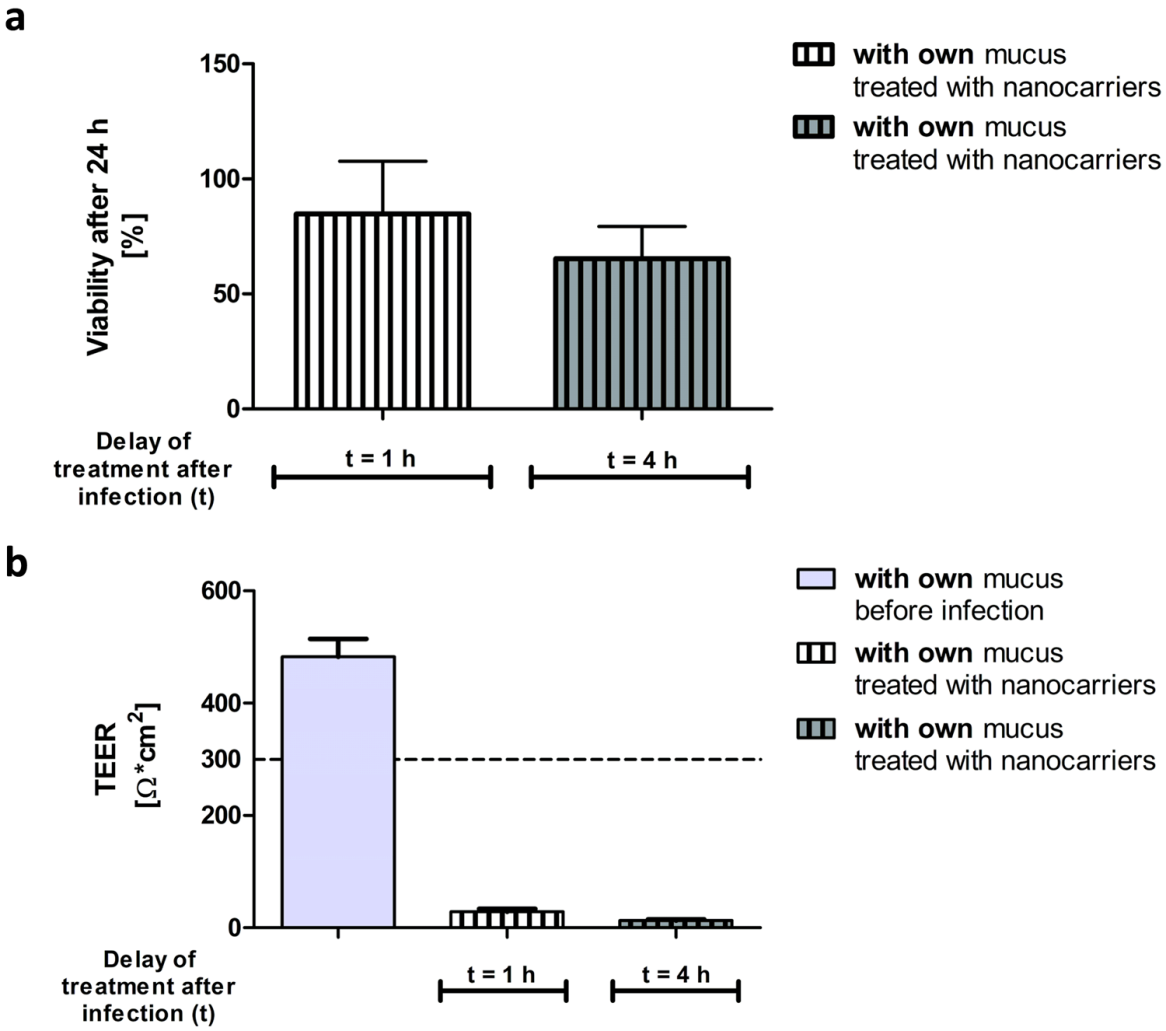
Viability and TEER of Calu-3 cells after infection. Viability (**a**) of Calu-3 cells was measured with an MTT assay. A decline in cell viability was visible. An early treatment (*t* = 1 h) with the antibiotic-loaded nanocarriers maintained 80% viability, whereas a later treatment (*t* = 4 h) resulted in only 50% viable cells 24 h after the infection. TEER (**b**) measurement before the infection showed good barrier properties (TEER ~ 500 Ω*cm^2^). However, even with an early treatment time point (*t* = 1 h), the barrier properties were lost when the cells were infected. Experiments were performed at least 3 times, and results are presented as mean ± SEM

During the development of the model, the well-known Calu-3 HTB-55 cells (also of bronchial origin) that can produce a mucus layer [45] were also used. Compared to the CFBE41o- cell, the Calu-3 cells were more sensitive to bacterial infection than the CFBE41o- cells. The MTT assay (Fig. 5a) showed that even with early antibiotic-nanocarrier treatment (*t* = 1 h), viability was reduced to 80%. Viability further declined to 50% when nanocarriers were only administered after 4 h. TEER measurements (Fig. 5b) before the infection showed that Calu-3 cells exhibit good barrier properties (TEER ~ 500 Ω*cm^2^). However, after 24 h of infection, the barrier integrity could not be maintained, even with early treatment (*t* = 1 h, TEER ~ 30 Ω*cm^2^). Compared to the CFBE41o- cells, the Calu-3 cell line was more vulnerable to the infection and did not endure bacteria’s presence for a long time without compromising barrier properties and a decline in cell viability. We, therefore, chose the more robust CFBE41o- cells as a preferable cell line for such in vitro model.

**Fig. 6:**
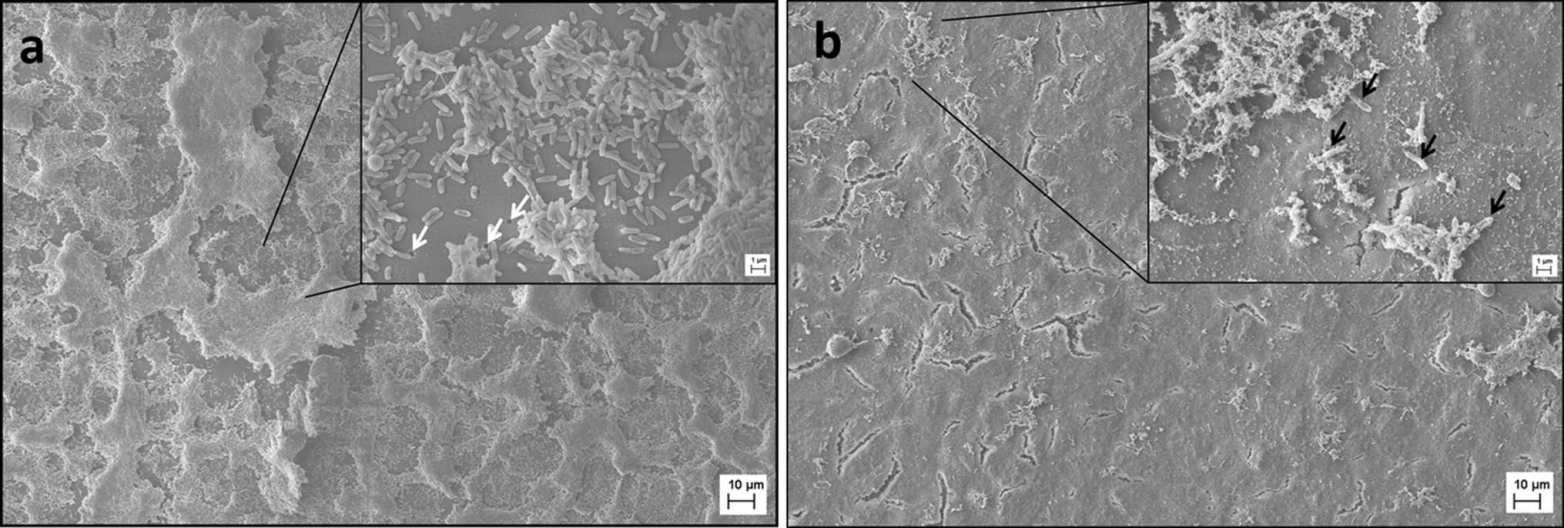
Scanning electron microscopy images of the co-culture without or with treatment. Cells were treated 1 h after the infection with blank KRB **a** or antibiotic-loaded nanocarriers **b**. Cells were fixed 24 h after the infection. KRB treatment resulted in the destruction of the epithelial cell monolayer and the formation of a biofilm covering the whole well. Pores of the Transwell® membrane are visible (white arrows). With nanocarrier treatment, the viability of epithelial cells could be maintained, and only single bacteria (black arrows) are visible on top of the still intact epithelial monolayer

Scanning electron microscopy micrographs of CFBE41o- cells treated with ciprofloxacin nanocarriers 1 h after the infection allowed for visual evaluation of epithelial and biofilm integrity, while drug-free KRB treated co-cultures served as control. When KRB was applied to the co-culture (Fig. 6a), no epithelial cells could be detected anymore after 24 h in co-culture with PAO1 biofilm*.* This is in agreement with the absence of TEER described before (Fig. 4b). Only a biofilm of the pathogen remained. Even the pores in the Transwell® membrane were visible (white arrows in the enlargement). In contrast, when drug-loaded nanocarriers were applied 1 h after the infection, the epithelial cell monolayer was still visible after 24 h (Fig. 6b). Only a few single bacteria (black arrows in the enlargement) embedded in a mixture of mucus and biofilm were visible on top of the bronchial cells.

**Fig. 7:**
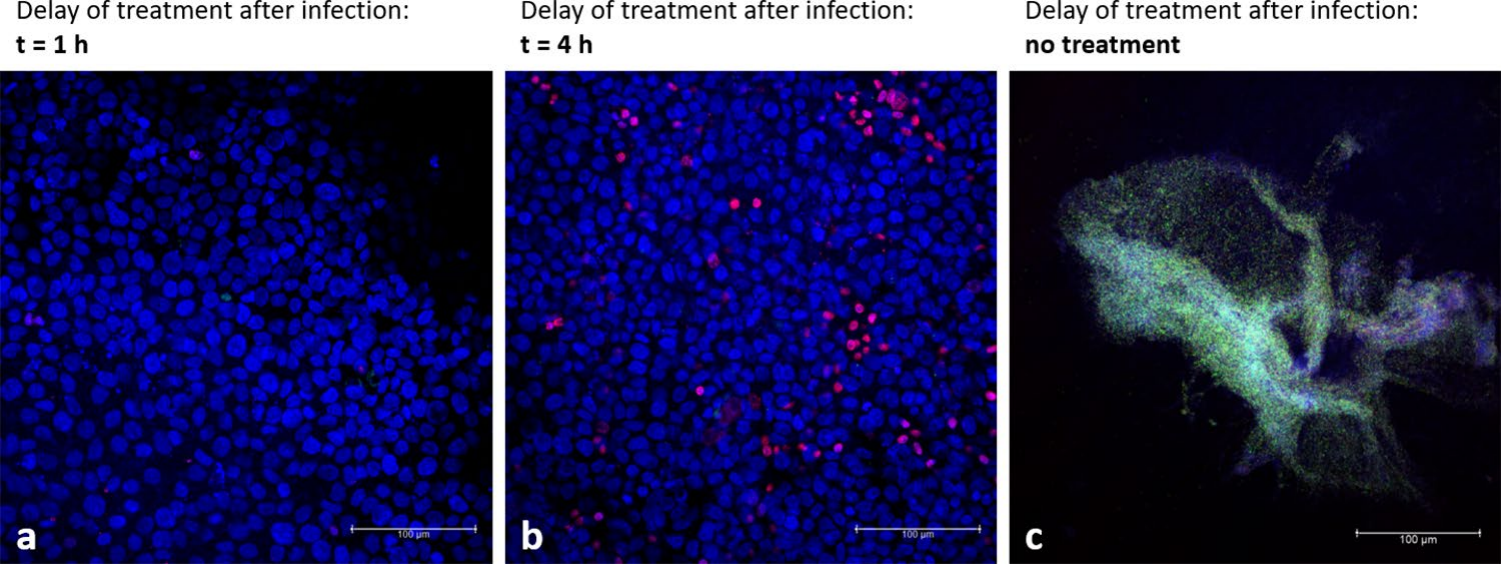
Representative confocal images of co-cultures after treatment or no treatment. The co-culture was fixed 24 h after the infection. When antibiotic-loaded nanocarriers were applied after 1 h (**a**), cells are still forming a confluent monolayer. Treatment after 4 h (**b**) resulted in increased cell death (red nuclei). No treatment (**c**) led to the destruction of the cell monolayer, and bacteria could build three-dimensional biofilm structures. DAPI (blue) was used to stain the nuclei of all cells and propidium iodide (red) to label dead cell nuclei; *P. aeruginosa* strain PAO1 carries a plasmid with GFP (green)

CLSM gave further insight into the status of the co-culture after antibiotic treatment at different time points. Figure 7 (a) depicts a confluent monolayer of the cells when nanocarriers were applied 1 h after the infection; here, no PI stained (dead) nuclei are visible. Later treatment (*t* = 4 h) increased cell death, as seen by the red-stained nuclei (Fig. 7b). No living bacteria (green) could be detected in Fig. 7(a, b). In contrast, when cells were treated with drug-free KRB, the cell monolayer was destroyed entirely. No cell nuclei were visible anymore. Since DAPI and PI bind to DNA abundant in bacterial biofilms, fluorescence imaging of the same was possible and revealed the typical three-dimensional structures (Fig. 7c) [46, 47].

### Efficacy of antibiotic-loaded nanocarriers against planktonic and biofilm *P. aeruginosa*

**Fig. 8:**
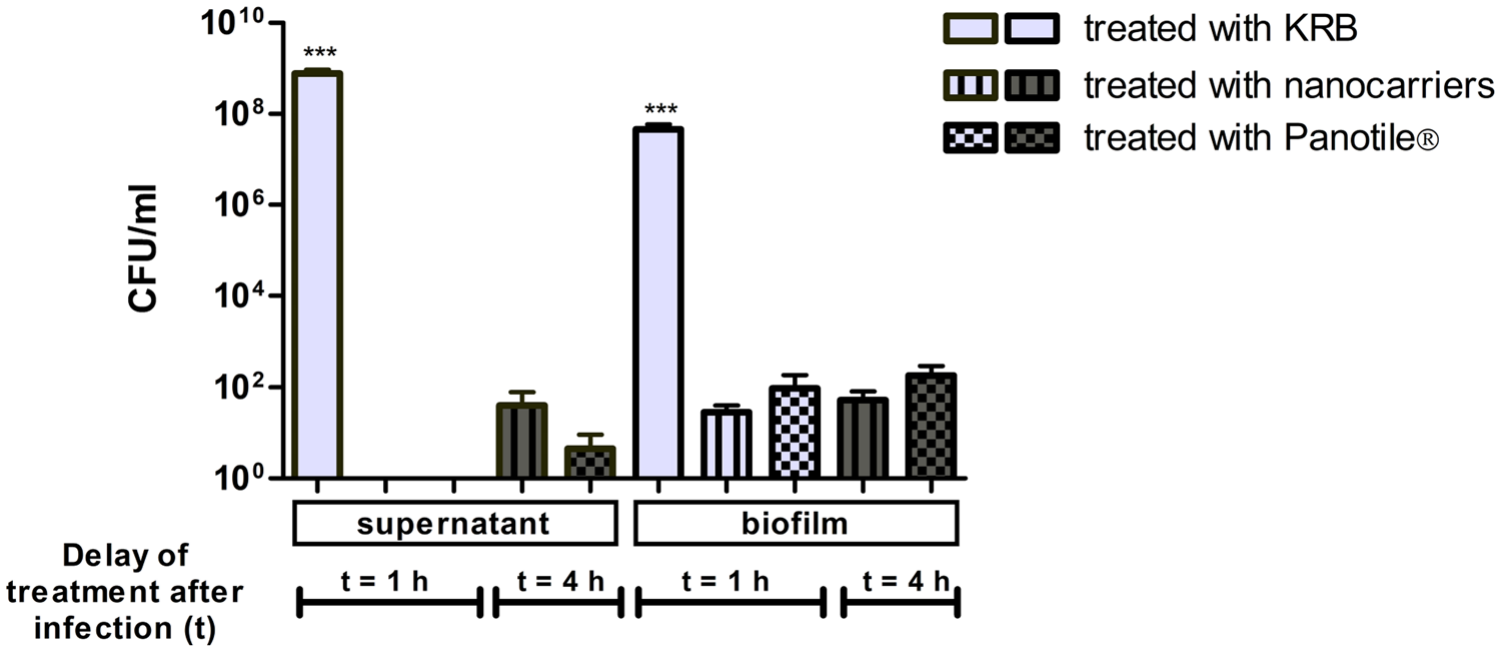
Remaining CFU in supernatant and biofilm. CFU/ml of PAO1 in supernatant and biofilm of KRB (control), antibiotic-loaded nanocarrier, or Panotile® treated cells was determined 24 h after the infection. All planktonic bacteria in the supernatant were eradicated when treatment was given 1 h after the infection. Later application of the treatment only increased the CFU/ml slightly. CFU count of biofilm fraction revealed that early treatment (*t* = 1 h) almost eradicates all biofilm bacteria. CFU doubles when the application of nanocarriers or Panotile® is only conducted at *t* = 4 h. Control wells (KRB only) showed a log 6 higher CFU/ml than wells that received antibiotic-loaded nanocarriers. Data are presented as mean ± SEM (*n* = 11 from 4 independent experiments). ***p < 0.0001

The efficacy of ciprofloxacin nanocarriers was compared to a commercially available ciprofloxacin product (Panotile® CIPRO ear drops). The same dosing schedules of 1 h and 4 h after the infection were chosen for the application of the antibiotic treatments or drug-free KRB as control, respectively. Planktonic and biofilm CFU/ml were determined 24 h after the infection accordingly (Fig. 8). At 24 h after the infection, the supernatants from cultures treated with the antibiotic-loaded nanocarriers or Panotile® solution 1 h after infection showed no bacterial growth. The later treatment (4 h after the infection) was not effective in eradicating all planktonic bacteria. For the biofilm fraction, nanocarriers’ treatment led to a log 6 reduction of CFU/ml compared to the control wells treated with KRB (2.8 × 10^1^ and 5.2 × 10^1^ CFU/ml for nanocarrier treatment after 1 h or 4 h vs. 4.6 × 10^7^ CFU/ml for KRB treatment). Similar to the ciprofloxacin nanocarriers, the Panotile® solution was able to reduce the bacterial burden. A log 5 reduction of CFU/ml could be achieved (9.5 × 10^1^ and 1.8 × 10^2^ CFU/ml for Panotile® treatment after 1 h or 4 h vs. 4.6 × 10^7^ CFU/ml for KRB treatment). The difference between the two treatments, however, did not yet reach statistical significance, indicating that there is potential and still room for further improvement in such a nanocarrier approach.

## Discussion

One might argue that research to improve in vitro models will not immediately provide a cure to better help patients in the clinic. However, the translational value of so-called complex in vitro models (CIVM’s) is the potential to eventually better predict the clinical performance of experimental drugs and delivery systems than currently used preclinical animal models. We here described a method for such a CIVM by manually transferring a *P. aeruginosa* biofilm to the apical surface of bronchial epithelial cells with a pipette. The physical forces involved with spreading the biofilm across the surface may explain the relatively smaller biofilm fractions found in Fig. 2b. According to the literature [39, 48], a minimum of 24 h of maturation on abiotic surfaces is required to provide an equally mature biofilm as if grown for 6 h on a biotic surface. Transferring the biofilm by pipetting resulted in multiple microcolonies compared to the large native biofilm colony in the 24-well plate (Fig. 3b and a, respectively). However, the biofilms biomass was not compromised during this process, as quantified by crystal staining (Fig. 3e), indicating the mature, preformed biofilm is transferred as micro-colonies while maintaining its structural integrity.

Lieleg et al. [49] demonstrated that biofilms have remarkable self-healing properties, and even though the manual transfer might have destroyed bigger biofilm colonies, the remaining microcolonies regain their former viscoelastic behavior. Interactions between bacteria and biofilm polymers will be re-established within minutes. The smaller microcolonies may even better reflect the actual in vivo situation, where the biofilm can be found only in parts of the lung tissue and is not covering the whole surface [50]. As suggested by Bjarnsholt et al. [51], microcolonies of biofilms might be more clinically relevant than the typically relatively large and dense biofilms of most in vitro models. Further in-depth characterization of transferred biofilms compared to native biofilms and planktonic bacteria is currently ongoing (Horstmann et al., in preparation).

Moreover, the biofilms observed on top of the mucus and cell monolayer in the scanning electron and CLSM micrographs in Fig. 2 are, in general, similar in density compared to the biofilms on top of the cell-only monolayer in the CLSM micrograph in Fig. 3c. Previously, Caldara et al. showed that mucins could inhibit bacterial adhesion to underlying surfaces and, therefore, could prevent bacterial colonization and biofilm formation [52]. However, in CF, *P. aeruginosa* tends to be non-motile [53], increasing its ability to build large, aggregated flocs in the presence of mucins [52]. While we had no access to mucus from CF patients, tracheobronchial mucus isolated from healthy volunteers undergoing elective surgery was used as a surrogate for establishing the model. We demonstrate biofilm formation on a cell monolayer with a mucus coating. Considering the bacteria used in our studies are already partly non-motile bound in a biofilm, this may have led to further rapid growth 24 h after transferring onto the cells in the presence of mucus, as observed in Figs. 6a and 7c with the destruction of the epithelial layer. Therefore, larger biofilm colonies may, in fact, harm the co-culture model developed.

Additional washing steps of the apical surface may improve the model protocol by reducing the planktonic bacteria, which are responsible for acute toxicity and cell death of the epithelial cells through to their rapid over-growth and the activation of the type 3 secretion system, a significant threat in acute *P. aeruginosa* infections [11, 31, 54]. It would furthermore allow more time for host–pathogen interactions between CFBE41o- cells and *P. aeruginosa* biofilm. However, while undoubtedly capable of further technical improvements, the experimental set-up, as described herein, already allowed safety and efficacy testing of aerosolized antibiotic nanocarriers against *P. aeruginosa* in vitro by maintaining ALI conditions during the whole course of the experiment.

The advantages of pulmonary drug delivery for lung diseases like CF and the benefits of administering antibiotic drugs like ciprofloxacin by this route have been discussed at the beginning of this paper. A dry powder inhaler and a liposomal formulation with ciprofloxacin have already been tested in phase III studies (RESPIRE I and II and ORBIT III and IV trials). However, disappointing results and inconsistency between the trials led to no market approval of the drug formulation by the FDA and a stop in developing the DPI for the time being [55–57]. This emphasizes the fact that further research on advanced aerosolizable ciprofloxacin-nanocarriers is of great importance.

Since pure ciprofloxacin suffers from low solubility, the preparation of a ciprofloxacin solution with similar drug content as the nanocarrier solution was not possible. To our knowledge, no pulmonary pharmaceutical product containing ciprofloxacin, and especially no nanoformulation, are on the market yet [56]. One of the few pharmaceutical products on the German market with ciprofloxacin already in solution is eardrops. In default of a better alternative, a market-approved commercial product—Panotile® CIPRO eardrops—was chosen to check the activity of free ciprofloxacin on biofilms, compared to the antibiotic nanocarriers. Therefore, the effect of the ciprofloxacin nanocarriers and commercial products on infected co-cultures were compared and further assessed by CLSM.

Even though CFU counting revealed that the treatment with the nanocarriers could not eradicate all biofilm grown bacteria, the lack of the bacterial signal in those images (Fig. 7a and b) can be explained by the fact that after 24 h, only a few bacteria (~ 50 CFU/ml, Fig. 8) remained in the whole sample. Moreover, the micrographs only represent approximately one-seventh of the total sample. CLSM data corroborates the previously discussed results regarding epithelial barrier function and cellular viability by showing that a later treatment increases the amount of red-stained nuclei, indicating cell death. Even holes in the cell monolayer are equivalent with a decrease in viability and TEER values.

Compared to the commercial product Panotile® CIPRO, our results with antibiotic-loaded nanocarriers tended to show some improved efficacy against bacterial biofilms (CFU/ml counts were only a third of the once treated with Panotile® CIPRO, see results section). Although the improvement of bacterial killing failed to reach significance, this observation supports the possibility of developing nanocarriers for improving penetration of mucus and bacterial biofilm but probably requiring new materials and technologies [21]. Also, nanocarriers with mucoadhesive properties and a prolonged residence time after deposition could be designed to release their cargo slowly. This would lead to drug levels above the minimum inhibitory concentration needed to eradicate bacteria for more extended periods in the pulmonary lining fluid. In contrast, a drug administered as a solution would be cleared from the lungs much more rapidly, either by mucociliary clearance or systemic absorption. The development of such an optimized nanocarrier was, however, beyond the scope of the present study.

## Conclusion

We here describe a protocol for cultivating preformed biofilms of *P. aeruginosa* on top of the CF cell line CFBE41o- at ALI conditions. The addition of human mucus to CFBE41o- cells before infection improved their barrier properties and survival during the infection.

To demonstrate the translational potential of such a complex in vitro model to assist in the fight against infectious diseases and the increasing threat of antimicrobial resistance, aerosolized antibiotic-loaded nanocarriers could be shown to efficiently reduce *P. aeruginosa* growing on the human bronchial cells both in planktonic and biofilm form. By allowing to optimize drug delivery across significant biological barriers (e.g., mucus, bacterial biofilms, epithelial cells), complex models of human tissues and organs in a state of diseases may significantly facilitate the preclinical development of innovative therapeutic modalities and their successful translation into the clinic.

## Supplementary Information

Below is the link to the electronic supplementary material.

Supplementary file1 (DOCX 17 KB)

## References

[1] Foundation CF. What is CF - About Cystic Fibrosis. https://www.cff.org/What-is-CF/About-Cystic-Fibrosis/. Accessed 11 Oct 2020.

[2] Haley CL, Colmer-Hamood JA, Hamood AN (2012). Characterization of biofilm-like structures formed by Pseudomonas aeruginosa in a synthetic mucus medium. BMC Microbiol.

[3] Heijerman H (2005). Infection and inflammation in cystic fibrosis: a short review. J Cyst Fibros.

[4] Lyczak JB, Cannon CL, Pier GB (2002). Lung infections associated with cystic fibrosis. Clin Microbiol Rev.

[5] Moreau-Marquis S, Coutermarsh B, Stanton BA (2015). Combination of hypothiocyanite and lactoferrin (ALX-109) enhances the ability of tobramycin and aztreonam to eliminate Pseudomonas aeruginosa biofilms growing on cystic fibrosis airway epithelial cells. J Antimicrob Chemother.

[6] Moreau-Marquis S, O'Toole GA, Stanton BA (2009). Tobramycin and FDA-approved iron chelators eliminate Pseudomonas aeruginosa biofilms on cystic fibrosis cells. Am J Respir Cell Mol Biol.

[7] Bjarnsholt T (2013). The role of bacterial biofilms in chronic infections. APMIS Suppl.

[8] Donlan RM, Costerton JW (2002). Biofilms: survival mechanisms of clinically relevant microorganisms. Clin Microbiol Rev.

[9] Prince A (2002). Biofilms, antimicrobial resistance, and airway infection. N Engl J Med.

[10] Høiby N, Bjarnsholt T, Givskov M, Molin S, Ciofu O (2010). Antibiotic resistance of bacterial biofilms. Int J Antimicrob Agents.

[11] Costerton JW (1999). Bacterial biofilms: a common cause of persistent infections. Science.

[12] Bhat PG, Flanagan DR, Donovan MD (1996). Drug diffusion through cystic fibrotic mucus: steady-state permeation, rheologic properties, and glycoprotein morphology. J Pharm Sci.

[13] Stewart PS, Costerton JW (2001). Antibiotic resistance of bacteria in biofilms. Lancet.

[14] Walters MC, Roe F, Bugnicourt A, Franklin MJ, Stewart PS (2003). Contributions of antibiotic penetration, oxygen limitation, and low metabolic activity to tolerance of Pseudomonas aeruginosa biofilms to ciprofloxacin and tobramycin. Antimicrob Agents Chemother.

[15] Di Gioia S, Trapani A, Castellani S, Carbone A, Belgiovine G, Craparo EF (2015). Nanocomplexes for gene therapy of respiratory diseases: targeting and overcoming the mucus barrier. Pulm Pharmacol Ther.

[16] Forier K, Raemdonck K, De Smedt SC, Demeester J, Coenye T, Braeckmans K (2014). Lipid and polymer nanoparticles for drug delivery to bacterial biofilms. J Control Release.

[17] Heijerman H, Westerman E, Conway S, Touw D, Doring G (2009). Inhaled medication and inhalation devices for lung disease in patients with cystic fibrosis: a European consensus. J Cyst Fibros.

[18] Scheuch G, Kohlhaeufl MJ, Brand P, Siekmeier R (2006). Clinical perspectives on pulmonary systemic and macromolecular delivery. Adv Drug Deliv Rev.

[19] Hadinoto K, Cheow WS (2014). Nano-antibiotics in chronic lung infection therapy against Pseudomonas aeruginosa. Colloids Surf B Biointerfaces.

[20] D'Angelo I, Casciaro B, Miro A, Quaglia F, Mangoni ML, Ungaro F (2015). Overcoming barriers in Pseudomonas aeruginosa lung infections: Engineered nanoparticles for local delivery of a cationic antimicrobial peptide. Colloids Surf B Biointerfaces.

[21] Ho DK, Murgia X, De Rossi C, Christmann R, Hüfner de Mello Martins AG, Koch M et al. Squalenyl hydrogen sulfate nanoparticles for simultaneous delivery of tobramycin and an alkylquinolone quorum sensing inhibitor enable the eradication of P. aeruginosa biofilm infections. Angew Chem Int Ed Engl. 2020;59(26):10292–6. 10.1002/anie.202001407. 10.1002/anie.202001407 PMC7317969 32243047

[22] Bayes HK, Ritchie N, Irvine S, Evans TJ. A murine model of early Pseudomonas aeruginosa lung disease with transition to chronic infection. Sci Rep. 2016;6(35838). 10.1038/srep35838 PMC5090221 27804985

[23] Baldan R, Cigana C, Testa F, Bianconi I, De Simone M, Pellin D et al. Adaptation of Pseudomonas aeruginosa in Cystic Fibrosis airways influences virulence of Staphylococcus aureus in vitro and murine models of co-infection. PLoS One. 2014;9(3). 10.1371/journal.pone.0089614 PMC3945726 24603807

[24] Bakker-Woudenberg IA, ten Kate MT, Guo L, Working P, Mouton JW (2002). Ciprofloxacin in polyethylene glycol-coated liposomes: efficacy in rat models of acute or chronic Pseudomonas aeruginosa infection. Antimicrob Agents Chemother.

[25] Lebeaux D, Chauhan A, Rendueles O, Beloin C (2013). From in vitro to in vivo models of bacterial biofilm-related infections. Pathogens.

[26] Russell WMS, Burch RL. The principles of humane experimental technique. Methuen; 1959.

[27] Ceri H, Olson ME, Stremick C, Read RR, Morck D, Buret A (1999). The Calgary Biofilm Device: new technology for rapid determination of antibiotic susceptibilities of bacterial biofilms. J Clin Microbiol.

[28] Elkhatib W, Noreddin A (2014). Efficacy of ciprofloxacin-clarithromycin combination against drug-resistant Pseudomonas aeruginosa mature biofilm using in vitro experimental model. Microb Drug Resist.

[29] Zemke AC, Shiva S, Burns JL, Moskowitz SM, Pilewski JM, Gladwin MT (2014). Nitrite modulates bacterial antibiotic susceptibility and biofilm formation in association with airway epithelial cells. Free Radic Biol Med.

[30] Moreau-Marquis S, Redelman CV, Stanton BA, Anderson GG. Co-culture models of Pseudomonas aeruginosa biofilms grown on live human airway cells. Journal of Visualized Experiments. 2010(44). 10.3791/2186. 10.3791/2186 PMC3185622 20972407

[31] Anderson GG, Moreau-Marquis S, Stanton BA, O'Toole GA (2008). In vitro analysis of tobramycin-treated Pseudomonas aeruginosa Biofilms on Cystic Fibrosis-derived airway epithelial cells. Infect Immun.

[32] Müller L, Murgia X, Siebenbürger L, Börger C, Schwarzkopf K, Sewald K (2018). Human airway mucus alters susceptibility of Pseudomonas aeruginosa biofilms to tobramycin, but not colistin. J Antimicrob Chemother.

[33] Gunday Tureli N, Tureli AE, Schneider M (2016). Optimization of ciprofloxacin complex loaded PLGA nanoparticles for pulmonary treatment of cystic fibrosis infections: Design of experiments approach. Int J Pharm.

[34] Gunday Tureli N, Tureli AE, Schneider M (2016). Counter-ion complexes for enhanced drug loading in nanocarriers: proof-of-concept and beyond. Int J Pharm.

[35] Günday Türeli N, Torge A, Juntke J, Schwarz BC, Schneider-Daum N, Türeli AE et al. Ciprofloxacin-loaded PLGA nanoparticles against cystic fibrosis P. aeruginosa lung infections. Eur J Pharm Biopharm. 2017;117:363–71. 10.1016/j.ejpb.2017.04.032. 10.1016/j.ejpb.2017.04.032 28476373

[36] Horstmann JC, Thorn CR, Carius P, Graef F, Murgia X, De Souza Carvalho Wodarz C et al. A custom-made device for reproducibly depositing pre-metered doses of nebulized drugs on pulmonary cells in vitro. Frontiers in Bioengineering and Biotechnology. 2021. 10.3389/fbioe.2021.643491. 10.3389/fbioe.2021.643491 PMC8096921 33968912

[37] Bruscia E, Sangiuolo F, Sinibaldi P, Goncz KK, Novelli G, DC. G. Isolation of CF cell lines corrected at DeltaF508-CFTR locus by SFHR-mediated targeting. Gene Ther. 2002 Jun;9(11):683–5. 10.1038/sj/gt/3301741. 10.1038/sj.gt.3301741 12032687

[38] Cozens AL, Yezzi MJ, Kunzelmann K, Ohrui T, Chin L, Eng K (1994). CFTR expression and chloride secretion in polarized immortal human bronchial epithelial cells. Am J Respir Cell Mol Biol.

[39] Moreau-Marquis S, Bomberger JM, Anderson GG, Swiatecka-Urban A, Ye S, O'Toole GA (2008). The DeltaF508-CFTR mutation results in increased biofilm formation by Pseudomonas aeruginosa by increasing iron availability. Am J Physiol Lung Cell Mol Physiol.

[40] Ehrhardt C, Collnot E-M, Baldes C, Becker U, Laue M, Kim K-J (2005). Towards an in vitro model of cystic fibrosis small airway epithelium: characterisation of the human bronchial epithelial cell line CFBE41o. Cell Tissue Res.

[41] Murgia X, Yasara H, Carvalho-Wodarz C, Loretz B, Gordon S, Schwarzkopf K et al. Modelling the bronchial barrier in pulmonary drug delivery: a human bronchial epithelial cell line supplemented with human tracheal mucus. Eur J Phar Biopharm. 2016. 10.1016/j.ejpb.2017.03.020 28373109

[42] Holloway BW, Morgan AF (1986). Genome organization in Pseudomonas. Annu Rev Microbiol.

[43] Peeters E, Nelis HJ, Coenye T (2008). Comparison of multiple methods for quantification of microbial biofilms grown in microtiter plates. J Microbiol Methods.

[44] Stepanovic S, Vukovic D, Dakic I, Savic B, Svabic-Vlahovic M (2000). A modified microtiter-plate test for quantification of staphylococcal biofilm formation. J Microbiol Methods.

[45] Fiegel J, Ehrhardt C, Schaefer UF, Lehr CM, Hanes J (2003). Large porous particle impingement on lung epithelial cell monolayers–toward improved particle characterization in the lung. Pharm Res.

[46] O'Toole G, Kaplan HB, Kolter R (2000). Biofilm formation as microbial development. Annu Rev Microbiol.

[47] Kostakioti M, Hadjifrangiskou M, Hultgren SJ. Bacterial biofilms: development, dispersal, and therapeutic strategies in the dawn of the postantibiotic era. Cold Spring Harbor Perspectives in Medicine. 2013;3(4):a010306-a. 10.1101/cshperspect.a010306. 10.1101/cshperspect.a010306 PMC3683961 23545571

[48] Klausen M, Heydorn A, Ragas P, Lambertsen L, Aaes-Jørgensen A, Molin S (2003). Biofilm formation by Pseudomonas aeruginosa wild type, flagella and type IV pili mutants. Mol Microbiol.

[49] Lieleg O, Caldara M, Baumgärtel R, Ribbeck K (2011). Mechanical robustness of Pseudomonas aeruginosa biofilms. Soft Matter.

[50] Bjarnsholt T, Jensen PØ, Fiandaca MJ, Pedersen J, Hansen CR, Andersen CB (2009). Pseudomonas aeruginosa biofilms in the respiratory tract of cystic fibrosis patients. Pediatr Pulmonol.

[51] Bjarnsholt T, Alhede M, Alhede M, Eickhardt-Sørensen SR, Moser C, Kühl M (2013). The in vivo biofilm. Trends Microbiol.

[52] Caldara M, Friedlander RS, Kavanaugh NL, Aizenberg J, Foster KR, Ribbeck K (2012). Mucin biopolymers prevent bacterial aggregation by retaining cells in the free-swimming state. Curr Biol.

[53] Mahenthiralingam E, Campbell ME, Speert DP (1994). Nonmotility and phagocytic resistance of Pseudomonas aeruginosa isolates from chronically colonized patients with cystic fibrosis. Infect Immun.

[54] Sadikot RT, Blackwell TS, Christman JW, Prince AS (2005). Pathogen-host interactions in Pseudomonas aeruginosa pneumonia. Am J Respir Crit Care Med.

[55] Bassetti M, Vena A, Russo A, Peghin M (2020). Inhaled liposomal antimicrobial delivery in lung infections. Drugs.

[56] Chorepsima S, Kechagias KS, Kalimeris G, Triarides NA, Falagas ME (2018). Spotlight on inhaled ciprofloxacin and its potential in the treatment of non-cystic fibrosis bronchiectasis. Drug Des Devel Ther.

[57] Bayer. Bayer Annual Report 2017 – Augmented Version 2018.

