# Supplementary material for: Testing of aerosolized ciprofloxacin nanocarriers on cystic fibrosis airway cells infected with P. aeruginosa biofilms
Source: Drug Deliv Transl Res. 2021 May 28;11(4):1752–65. doi: 10.1007/s13346-021-01002-8 (PMC8236054; doi:10.1007/s13346-021-01002-8)
Supplement: Supplementary file 1 — Supplementary file1 (DOCX 17 KB) [file 13346_2021_1002_MOESM1_ESM.docx]

Supplementary material to Juntke et al.:

**Table 1** Particle size measurement of resuspended and nebulized nanocarriers

| **Sample** | **Measurement** | **Z-Average size**  **[d.nm]** | **Polydispersity Index (PdI)** | **Average ± SEM**  **[nm]** |
| --- | --- | --- | --- | --- |
| Nanocarrier in KRB | 1 | 329,7 | 0,434 | 374.2 ± 40.77 |
|  | 2 | 337,2 | 0,449 |  |
|  | 3 | 455,6 | 0,452 |  |
| nebulized Nanocarrier in KRB | 1 | 318,5 | 0,451 | 320.8 ± 1.577 |
|  | 2 | 320,0 | 0,446 |  |
|  | 3 | 323,8 | 0,470 |  |

_Standard error of the mean (SEM)_

**Table 2** Recovery rate of nebulized nanocarriers measured with UHPLC

| **Sample** | | **AUC** | **Konz. 1:100**  [µM] | **Konz.**  [µM] | **Average ± SEM** [µM] |
| --- | --- | --- | --- | --- | --- |
| 1 | 1.1 | 4,6293 | 2,9386 | 293,86 | 227,7 ±53,87 |
|  | 1.2 | 1,7434 | 1,2094 | 120,94 |  |
|  | 1.3 | 4,201 | 2,6820 | 268,20 |  |
| 2 | 2.1 | 4,7676 | 3,0215 | 302,15 | 206,7 ± 68,90 |
|  | 2.2 | 3,8131 | 2,4496 | 244,96 |  |
|  | 2.3 | 0,9413 | 0,7288 | 72,88 |  |
| 3 | 3.1 | 2,69 | 1,7766 | 177,66 | 269,1 ± 45,89 |
|  | 3.2 | 5,0937 | 3,2169 | 321,69 |  |
|  | 3.3 | 4,8647 | 3,0797 | 307,97 |  |
| **Average ± SEM** [µM] | |  | | | 234,5 ± 30,0 |
| **Recovered amount** [%] | |  | | | **67,0 ± 8,6** |

_3 samples (each 1 ml; concentration of 350 µM) were prepared; 200 µl of each sample were nebulized in separate wells of a 12-well plate; triplicates were made_

|  |  |  |
| --- | --- | --- |
|  |  |  |
|  |  |  |
